# Reviewed article: Farias BO, Araújo LF, Tavares NHC, Oliveira BBR, Vasconcelos MSL, Machado SP. Intersectionality of gender, race/skin color and dental loss in adults: cross-sectional study with data from the 2019 Brazilian National Health Survey. Epidemiol Serv Saude. 2025:34;e20240524

**DOI:** 10.1590/S2237-96222025v34e20240524.b

**Published:** 2025-08-04

**Authors:** Edgar Oshiro

**Affiliations:** Secretaria de Estado de Saúde de Mato Grosso do Sul, Escola de Saúde Pública

**Table:** 

Rating	Excellent	Good	Average	Below average	Poor
Interest			✓		
Quality		✓			
Originality			✓		
Overall		✓			

**Table:** 

Questionnaire	Yes	No	Not applicable
Does the manuscript contain new and significant information to justify publication?	✓		
Does the Abstract (Summary) clearly and accurately describe the content of the article?	✓		
Is the problem significant and concisely stated?	✓		
Are the methods described comprehensively?	✓		
Are the interpretations and conclusions justified by the results?	✓		
Is adequate reference made to other work in the field?	✓		

## Manuscript Structure

Length of article is: Adequate

Number of tables is: Adequate

Number of figures is: Adequate

Please state any conflict(s) of interest that you have in relation to the review of this paper (state “none” if this is not applicable): None

## Comments

O estudo tem um bom delineamento com informação da amostra, resultados e a análise estatística utilizada para a proposta de estudo.

O resumo apresenta estruturado, porém algumas palavras-chave não são descritores em ciências da saúde. Sugere-se a alteração para nterseccionalidade/Intersectionality; Extração dentária/tooth extraction, Iniquidade social/social inequity.

Sugere-se outros estudos populacionais comparativos quanto a mulheres serem mais suscetíveis a perda dentária, com referências as mulheres negras, que possam trazer fatores envolvidos na questão do gênero.

